# Pedagogies of engagement in science: A comparison of PBL, POGIL, and PLTL*

**DOI:** 10.1002/bmb.20204

**Published:** 2008-07

**Authors:** Thomas Eberlein, Jack Kampmeier, Vicky Minderhout, Richard S Moog, Terry Platt, Pratibha Varma-Nelson, Harold B White

**Affiliations:** ‡ School of Science, Engineering, and Technology, Pennsylvania State University-HarrisburgMiddletown, Pennsylvania 17057-4898; § Department of Chemistry, University of RochesterRochester, New York 14627-0216; ¶ Department of Chemistry, Seattle UniversitySeattle, Washington 98122; ∥ Department of Chemistry, Franklin and Marshall CollegeLancaster, Pennsylvania 17604; ‡‡ Department of Biology, University of RochesterRochester, New York 14627-0211; §§ Department of Chemistry, Northeastern Illinois UniversityChicago, Illinois 60625-4699; ¶¶ Department of Chemistry and Biochemistry, University of DelawareNewark, Delaware 19716-2522

**Keywords:** PBL, POGIL, PLTL, active-learning, student-centered, pedagogy

## Abstract

Problem-based learning, process-oriented guided inquiry learning, and peer-led team learning are student-centered, active-learning pedagogies commonly used in science education. The characteristic features of each are compared and contrasted to enable new practitioners to decide which approach or combination of approaches will suit their particular situation.

## INTRODUCTION

Problem-based learning (PBL),^1^ process-oriented guided inquiry learning (POGIL), and peer-led team learning (PLTL) represent three student-centered pedagogies in science that have received wide attention and NSF support in the past two decades. All are motivated by a call for change in the way we teach that fundamentally recognizes the way people learn [1]. All have much to offer, but each has particular emphases and applicability. For faculty interested in adopting active-learning strategies, the “PX_n_L” pedagogies provide a rich array of options, but may generate confusion due to their mix of shared and contrasting features. Our goal here is to describe, compare, and contrast the characteristics of these three pedagogies with the recognition that each is evolving in practice and that hybridization among them and with other approaches occurs frequently. As long-time practitioners of one or more of these pedagogies in a variety of higher education settings, we appreciate that the suitability of one or the other for particular situations will depend on the student audience, facilities, instructional goals, personal preferences, and available resources. We hope that the information and perspectives that we offer will stimulate interested colleagues to learn more about those approaches that seem well-suited to their situation and needs.

Undergirding each of the PX_n_L pedagogies are the tenets of *social constructivism* [2]. As the term is typically used in educational contexts, constructivism recognizes that knowledge is constructed in the mind of the learner by the learner [3]. Social constructivism implies that this “building” process is aided through cooperative social interactions. Simultaneously, it implies that lecture-only methods of teaching do not take optimal advantage of the unique characteristics of the educational milieu, in which many students are engaged in a mutual effort to both master course content and to learn how to learn. Because the human mind has limitations on the rate and amount of new information it can accurately assimilate and comprehend, any strategy that attempts to transfer knowledge more or less directly from teacher to student—“teaching by telling” is ineffective for many if not most students [4]. As cognitive load increases, the need for student engagement increases. Students must actively build for themselves a workable understanding of sophisticated concepts, and must be engaged in developing their own higher-order thinking skills. The PX_n_L pedagogies intentionally create learning environments that stimulate students to construct a robust understanding of concepts.

We will first provide a brief description of the three pedagogies and the theoretical bases for each. A detailed comparison follows in which the similarities and differences among the pedagogies are fleshed out. This comparison amplifies the entries in Table 1 that summarize the content and serve as the centerpiece of this article.

**Table 1:** Comparison of problem-based learning, process-oriented guided inquiry learning, and peer-led team learning

			Pedagogy		
Points of comparison			PBL	POGIL	PLTL
A. Fundamental aspects	1. Purpose		To promote higher-order thinking skills; to help students learn to reason though problems, instead of using algorithmic approaches; to build conceptual understanding through active engagement with the material; to foster growth in teamwork and collaborative problem-solving skills		
	2. Theoretical basis (“why it works”)		Constructivist ideas of Dewey and Piaget	Constructivism and the “learning cycle”	Constructivism and “zone of proximal development”
	3. Particular emphases		Need-to-know learning	Learning cycle format	Peer-led learning team
B. Classroom characteristics: Set-up; Roles and responsibilities; Materials and methods	1. Are lectures retained?		Sometimes	No	Yes
	2. Course format or supplemental		Course format	Course format	Supplemental, but integral to course
	3. Group problem solving sessions		During normal class hours; usually, all groups in the same room		Extra sessions usually held outside normal class hours; each group in a separate room
	4. Is the course grader present?		Yes	Yes	No
	5. Group work facilitation:	a. By whom?	Instructor (±peer facilitators)	Instructor	Peer leaders
		b. What do they do?	Instructor circulates, peer tutors promote interaction within a group	Instructor circulates among groups intervening only if necessary	Peer leader stays with one group, promotes group interaction
		c. Training?	Facilitator training course, or pre-semester training sessions	Attend POGIL workshops; find materials at POGIL website	Orientation, weekly faculty/leader meetings; leader training course
	6. Nature of the problems/in-class student work sessions:	a. Problem types	Complex, open-ended, real world, deliberately vague (sometimes)	Structured by learning cycle: exploration, invention, application	Similar to most challenging examination problems; structured for group work
		b. Duration	Varies; can range from a single class to an entire semester	One activity lasts one period; unfinished portions are homework	One session lasts for 1–2 hr, with many problems per session
		c. Problem sources	Primary literature; reworked stories of case-based issues	Published workbooks; problem sets adapted from workbooks by the instructor; problem sets of the instructor's own creation; websites	
	7. How are “concepts” treated?		Problems drive concept discovery on a need-to-know basis	Develop concepts through group work, reinforce w/ application	Probe and apply concepts introduced in text, lecture, and homework
	8. In-class textbook use?		Textbook, if any, used as one of many resources	Textbook not used in class; reading done after group work except in upper division courses	Textbook is resource for problem solving work sessions
	9. Groups	a. Ideal group size	4–5 (some use 8–10)	3–5	6–8
		b. Permanent groups	Yes	No	Yes
	10. Students' responsibilities	a. Responsibility of individuals to the group	Students must do individual research to bring back to group	Students must each play their assigned roles to ensure effective group work	Students must each prepare adequately to make worthwhile contributions to the group effort
		b. Group's responsibility to its members	Individuals have a responsibility to the group, but the group also has a responsibility to each individual to ensure a shared understanding of the concepts developed and/or reinforced in the group work session		
	11. Class size limits (“scalability”)		Ideal class size <30 students. Managing larger groups is doable, and similar to PLTL	Ideal class size <30 students. Many techniques available for adapting to large classes	No restrictions on class size. Limited only by peer leaders' and meeting rooms' availability
C. Out-of-class: Preparation; Follow-up; Grading	1. Preparation for class	a. By instructor	Prepare appropriate problems; prepare tutors for facilitation	Prepare group activity and quiz; anticipate problems	Prepare workshop materials; prepare peer leaders for facilitation
		b. By students	Students individually gather information for addressing “learning issues,” and prepare to share their findings with group	Complete previous activity and related reading; prepare for quiz, or complete assignments in upper division	Students complete all related reading and homework relevant to upcoming workshop
		c. By facilitators	Weekly tutor meeting with instructor; plus separate course	Instructor is the facilitator	Weekly peer leader meeting; leaders complete workshop as though they were students
	2. Homework	a. Types of problems	Students conduct research for addressing “learning issues”	Exercises and problems related to group work; textbook problems	(Homework from lecture class)
		b. Use of textbook	Multiple resources are necessary, and usually include the text, internet, primary literature, review articles, and seeking out experts	Textbook reading in intro courses done after the group work has led to the formation of an important concept, variable in upper courses	Textbook is for reading and homework problems, as in any lecture course; completed before the workshop
	3. Grading	a. In-class group work	Major effect on course grade from attendance, participation, preparation, and attitude	Some credit usually assigned for group work, attendance, and participation	Workshop attendance and participation usually does not have direct effect on grade
		b. Tests	Tests can include group effort	Tests usually involve only individual work	
D. Miscellaneous	1. Proven outcomes		PBL has most often been used in medical schools; data on outcomes are harder to come by	Decreased DFW rates, increased proportion of quality (ABC) grades; increase persistence to higher-level classes; no reduction in standardized test scores; other benefits	
	2. Benefits for peer facilitators		Authentic teaching experience helps leaders with their own learning; develops leadership skills	N/A	Authentic teaching experience helps leaders with their own learning; develops leadership skills
	3. Required resources		Recruitment, training, and compensation for (peer) tutors	Same resources required as for ordinary lecture courses	Recruitment, training, and compensation for peer leaders; need rooms for group work

## DESCRIPTIONS OF THE PEDAGOGIES

### Problem-Based Learning [5]

PBL originated in medical education as an alternative to information-dense lectures given to large classes [6]. Students working cooperatively in groups of eight to 10 with a facilitator explored the basic medical sciences, for example, biochemistry, physiology, anatomy, endocrinology, and microbiology in the integrated context of actual patient cases (the problems). PBL is driven by the premise that basic science concepts will be understood and remembered longer when they are learned, discussed, and applied in a practical, real-world context. An essential and distinctive feature of the approach is that problems come first and introduce content, rather than problems following a presentation of facts and concepts. Students learn on a need-to-know basis by group-directed exploration with the idea that they gain experience on the way to becoming self-directed learners.

Although the basic premises of the medical school PBL model have been retained, the source of problems and the classroom structure required adaptation to the college and university settings [7–9]. In the latter settings, students work cooperatively in smaller groups of four or five students to solve complex, open-ended problems that are based typically on real-world situations, but usually not medical cases. For example, in one model, students attempt to read an article from the primary literature on their own [10,11]. At the next class meeting, group members collaborate to define *learning issues*—words, concepts, or procedures—which they will need to learn about before they can truly understand the article. Learning issues (sometimes called “learning objectives”) are ranked in order of perceived importance and assigned to group members to look up before the next class. After several iterations of this process, students might demonstrate their understanding by completing a specific assignment such as writing a 200-word abstract of the article, or constructing a concept map based on the article.

Often instructors create PBL problems by reworking articles into stories about unresolved and messy real-world situations that require integrating multiple disciplinary perspectives even though this may not lead to a unique solution. In the undergraduate setting, group work is facilitated by the instructor sometimes assisted by near-peers who have taken the course before. PBL has origins as a lecture-less pedagogy. In practice, however, many instructors intersperse lectures with PBL problems stretched out over one or more class periods. Recently, the instructional approaches of PBL have been viewed as a way to actively encourage students to think like scientists and as a prelude to undergraduate research [12].

### Process Oriented Guided Inquiry Learning [13]

POGIL, like PBL, was designed to replace lectures in the classroom and thereby involve students in discussing the course material, rather than just hearing about it. Students work in self-managed teams during class on specially designed materials. These activities consist of a series of carefully crafted questions (the “guided inquiry”) that generally follow the three-phase “learning cycle” approach [14–17] which includes an exploration phase, a concept invention phase, and an application phase. In the “exploration” phase, students examine a “model,” search for patterns within it, and attempt to extract meaning from it. The model consists of any combination of pictures, tables, equations, graphs, prose, or other types of information. Often, the questions lead students to test hypotheses or explain the patterns and relationships found in the model. Next, in the “concept invention” (or “term introduction”) phase, a specific concept or relationship emerges and a term may be introduced to describe the newly developed concept or relationship. Alternatively, rather than being invented, the concept may be more fully developed or generalized during this phase. In this case, the phase is referred to as “concept formation” rather than “concept invention.” Finally, the “application” phase gives students the opportunity to extend and apply the concept to new situations, augmenting their understanding of the concept. The sequence of questions in POGIL materials are carefully devised to help students progress properly through the phases, to guide them toward appropriate conclusions, and to develop desired process skills, such as problem solving, deductive reasoning, communication, and self-assessment. Examples of POGIL materials can be found at the POGIL website [13].

In POGIL, the instructor serves as a facilitator to assist groups in the learning process and does not answer questions that students should be able to answer on their own. Students are assigned specific roles such as manager, recorder, reflector, technician, and presenter. The facilitation planned by the instructor and the roles that the students fulfill enable the development of process skills beyond what is addressed within the activity itself [16,18,19]. In some ways, this is similar to the team-based learning approach developed by Michaelsen *et al.* [20,21].

The POGIL approach has been used successfully in all of the typical areas of undergraduate chemistry, as well as biology, physics, mathematics, computer science, engineering, environmental science, education, and in high-school settings (predominantly in chemistry and biology). POGIL approaches can also be used for developing laboratory experiments [22–25]. Although POGIL has been used most extensively in small classes, it has been adapted for classes as large as several hundred with much success [26,27].

The essential elements for POGIL implementation are the use of small, self-managed groups of students; the role of the instructor as facilitator; the use of specially designed activities that generally follow the learning cycle paradigm; and the emphasis on development of process skills in addition to mastering course content.

### Peer-Led Team Learning [28]

Unlike PBL and POGIL, PLTL supplements, but generally does not replace, lecture time with group work sessions called “workshops.” There are no inherent restrictions on the size of the class. Under the PLTL model, undergraduate students who have done well in the class previously are recruited and trained as workshop leaders—“peer leaders”—who guide the efforts of a group of six to eight students. These peer-led groups meet weekly (separate from the lecture and the instructor) to work together on problems that are carefully structured to help the students build conceptual understanding and problem-solving skills. There are no answer keys for either the students or the peer-leaders; the emphasis is on learning to find, evaluate, and build confidence in answers. Simultaneously, the workshops and the peer leaders provide a supportive environment that helps each student participate actively in the process of learning science. Thus, PLTL offers a mix of active-learning opportunities for students and a new role for undergraduate peer leaders that is appropriate for their stage of development. PLTL has been used successfully in courses in chemistry, biology, physics, math, computer science, and engineering. In practice, the weekly workshop replaces traditional recitation sections led by graduate teaching assistants or faculty. Although most peer leaders are undergraduates, many graduate students with appropriate training have also worked effectively and enthusiastically in that role.

Through many years of workshop evaluations, the developers of PLTL identified six “critical components” [29] vital to ensuring the success of a PLTL program.

1. It is essential that the workshops are closely integrated with the course and all its elements.
2. Faculty teaching these courses must be actively involved with the workshops and with the peer leaders.
3. Peer leaders are students who have taken the course, who have good people skills, and who are well trained and supervised in facilitating small-group collaborative-learning sessions.
4. Workshop problems must be appropriately challenging and designed for use in collaborative group learning settings.
5. Organizational arrangements must ensure adequate and appropriate rooms for conducting workshop sessions.
6. Institutional and departmental support of innovative teaching methods is essential, including logistical and financial support.

## THEORETICAL BASES FOR THE PX_n_L PEDAGOGIES

Being the oldest of the PX_n_L pedagogies, PBL has evolved and diversified the most in its practice [30,31]. Although hybridization with other approaches has occurred, it demands more student independence than PLTL or POGIL. The originators of PBL in medical schools in the 1960s certainly had a sense of what did or did not promote learning [6,32]; however, they were not motivated by educational theory. Only more recently has learning theory been applied to the practice of PBL [33]. The constructivist ideas of Dewey [34] and Piaget [35] underlie much of today's PBL practice. From that perspective, instructors engage students with new challenging experiences and guide them to use those experiences to build and construct their own meaning and understanding.

Although PLTL and POGIL also have constructivist underpinnings, they were motivated more by specific concepts in learning than PBL. PLTL emphasizes the social aspects of learning developed by Vygotsky [36,37], in which the peers are often better catalysts for learning than the superiors [29]. Vygotsky argued that learning is essentially social and that there is a gap (the zone of proximal development or the ZPD) between learning outcomes produced in isolation and the level of potential development that can be achieved through collaboration with capable peers. PLTL situates students in their ZPD, as does PBL, by presenting challenging problems that they cannot solve easily on their own, but can accomplish by interaction with the members of the workshop team [38]. Thus, PLTL draws students into their ZPD—an area of learning gains they can achieve, but only with help—by having them work together on problems in groups in the instructor's absence, but with facilitation by a near peer who has successfully completed the course previously [39].

POGIL, like PLTL and PBL, uses students working together in groups, and therefore emphasizes the social aspects of learning. However, the POGIL groups have greater structure due to the assigned roles. This promotes the positive interdependence and accountability cited by Johnson *et al.* [40] and also provides opportunities for development of specific process skills through the student roles and the interactions between them.

The learning cycle that guides the structure of POGIL activities is derived from the mental functioning model proposed by Piaget [41]. Piaget identified several factors in the development of cognitive reasoning and suggested that two factors were essential for cognitive growth. Specifically, students must connect to their prior knowledge or past experience and they must experience a cognitively challenging situation. These ideas were incorporated into the learning cycle devised by Karplus and others, who developed materials for the Science Curriculum Improvement Study [42]. Later, these ideas were brought to the attention of the higher education community by, among others, Lawson [14] and Abraham and Renner [43].

## CLASSROOM CHARACTERISTICS

The PX_n_L pedagogies all focus on students discussing course content in small groups. Thus, the ideal physical setup of a classroom with chairs around circular or hexagonal tables and lots of blackboard space contrasts with a typical lecture classroom where seats or desks are often bolted to the floor facing forward, with writing boards or a slide projection screen at the front of the room. Despite the similarity in physical appearance associated with working groups in PX_n_L classrooms, the structure of the group activities differ with respect to the student tasks and role of the instructor.

For POGIL, group work takes the place of lecture time, but the instructor is still present to administer quizzes, distribute and collect materials, monitor progress, and intervene with groups that need guidance [16,19]. Occasionally, if many groups are struggling, the instructor may find it beneficial to insert a “mini-lecture” to clarify content and re-engage the students. Using assigned roles for students enables the groups to take much of the responsibility for learning the material. The role of the instructor-as-facilitator is to help students understand that they already possess the background and the reasoning skills necessary to develop new concepts and solve unfamiliar problems [44]. Often, instructors will respond to student questions by asking further questions. They monitor student progress toward meeting learning goals, and—if that progress is unsatisfactory—they make decisions about what form an intervention should take in order to ensure that the learning goals are met. Instructors help students to perform at a higher level than they could without the facilitation, but do not do the work for the students. They share responsibility with the group manager and reflector for maintaining constructive group dynamics. Instructors must also make decisions about the timing of students' oral reports of their results, which brings closure to any given portion of the activity, allows students to validate their answers, and maintains the pace of the class at an acceptable rate.

The structure of the classroom experience in advanced courses may be different. For example, in an upper-level biochemistry course, many of the fundamental concepts have already been developed in previous coursework. Students are held accountable for these concepts and may be asked to complete an assignment prior to class that serves to remind them of the prerequisite knowledge. In this way more class time is devoted to the concept formation phase and application phase of the learning cycle. Assigned homework is used for additional application experience. One example of this approach has recently been published [18].

Superficially, a PBL classroom might look like a POGIL classroom in that both have groups of four or five students and the instructor is present. (This is different than the original PBL model in medical schools where larger groups of eight to ten met separately with assigned expert tutor facilitators.) Because PBL problems are conceptually complex with few and rather open-ended prompting questions, students must generate the questions that guide learning. They must pursue these questions outside of class and bring back information to the group. As a consequence and in contrast to POGIL, the issues being discussed at one time in different PBL groups can be quite different. It is expected that different students will be learning different things in association with the core content objectives. Because groups can get off track, peer facilitators are especially helpful in classes with students having their first PBL experience [45]. The classroom role of the instructor is much the same as for POGIL except that he or she often joins groups for short times to facilitate discussions. Originally, PBL was intended to replace lectures in the medical school setting; however, many PBL courses now include lectures. The ideal PBL classroom has resource books and wireless laptop computers for students to look up information during class time. In many cases, instructors write or adapt the PBL problems they use.

By contrast to PBL and POGIL, PLTL retains lectures and has groups meet at separate times in different places. The instructors do not attend the workshop sessions because their presence perturbs the group interactions. Typically, PLTL groups have six to eight members and thus are larger than PBL or POGIL groups. Peer leaders manage the group dynamics and facilitate the collaborative problem-solving activities of the PLTL groups. Facilitating group discussion is an acquired skill for most workshop leaders and thus they need to be instructed in the art of questioning, the power of discussion and debate, and the principles and practice of collaborative and cooperative learning. Usually this is accomplished in a course taught by a teaching/learning expert in cooperation with the content instructor. When peer facilitators are used in PBL classes, similar preparation is needed [46].

Workshop leaders for PLTL and the peer facilitators sometimes used in PBL are recruited from the pool of students who have taken the course before, done well, and like the instructional format; but this is not enough. Good workshop leaders also have good interpersonal skills. Workshop leaders usually get paid for their service, and so financial resources are normally needed to implement the PLTL model. If funding is a problem, schools can offer course credit in place of a stipend. Alternatively, being a workshop leader may satisfy a service-learning requirement for graduation at some schools and at others both stipend and credit may be offered [45,46].

Typically, the instructor assigns students to PBL or PLTL groups at the beginning of the semester and group membership does not change during the course. In contrast, the composition of POGIL groups can change and the assigned roles rotate among group members.

## NATURE OF THE PROBLEMS AND ACTIVITIES

Although learning goals of the PX_n_L pedagogies have much in common, the nature of the classroom activities differ considerably among them. PBL uses complex problems, often presented as multistage, progressive-disclosure cases or stories, which may represent real-world dilemmas or controversies. They are distinctly interdisciplinary or at least integrate several topics, whereas PLTL and POGIL problems have a more specific disciplinary and conceptual focus. Resolution of PBL problems requires students to define the problem, identify information they need to acquire, and apply it in order to resolve the problem. The problem itself can take a single class period or span an entire semester [47]. Typically the first stage of a PBL problem is open-ended and intentionally vague so that students review what they already know and consider a variety of possibilities. Subsequent stages provide more information that serves to narrow the options and introduce additional things to consider. Ideally, resolution requires a decision based on careful analysis and reasoned assumptions. A PBL problem usually involves the integration of concepts, rather than focusing on a particular concept. It encourages problem-solving strategies and relies heavily on student initiative to locate resources and use the information they find. Because group progress in each successive class depends on every group member bringing new information to the discussion, PBL encourages students to be responsibly prepared and to attend class regularly. PBL problems that engage students' interest and clearly relate to the discipline help reinforce these behaviors. The nature of PBL makes it difficult to publish PBL activities because students searching for information would access and thereby short circuit the desired learning process. Although there are some books [48] and a password-protected PBL Clearinghouse for problems [49] that can be used “as is” or serve as models for writing new problems, PBL instructors often write their own.

In contrast to PBL, PLTL and POGIL activities are designed to be completed during class time. A PLTL session involves students working on a coherent set of problems designed by the course instructor to help students develop and internalize their understanding of key concepts and build problem-solving skills. They need to be suitable for group work and more challenging than typical end-of-chapter drill problems. For example, consider a case in which we are trying to help students understand how concentrations change with time and the concept of equilibrium for a reversible chemical reaction, A ⇄ B. Students in a PLTL workshop will have read text, listened to lectures and worked some problems on kinetics before coming to the workshop. In workshop, the concentrations of A and B are simulated with pennies and the reaction is modeled in successive exchanges by passing a percentage of the A pennies from student A to student B and vice versa. The students record the number of A and B pennies after each exchange (corresponding to a time interval) and ultimately construct a plot of concentration versus time. Different pairs of students are assigned different percentages of pennies for the forward and back reactions and the results of different simulations are compared and discussed.

PLTL problems assume that the students have completed the preliminary work of reading the text, studying the lectures and working homework problems from the book. Workshop problems are usually multistep, providing opportunities for discussion, visualization, and building understanding piece-by-piece. The leader's role is to facilitate the student–student discussion of the problems and the related concepts that will simultaneously lead to better understanding of the ideas and their application to finding answers to the problems. Although peer leaders will have worked through the problems in advance of the workshop, they are not given answer keys [29]. Prentice-Hall has published PLTL workshop books for various chemistry courses and workshop problems are now available in several other areas [50–52]. A broad array of workshop materials can be obtained from the PLTL website [28].

POGIL materials for introductory courses assume no prior knowledge of the topic of the day (other than the concepts already developed in the course) and thus are distinctly in contrast with PLTL problems. Many POGIL classes will begin with a short quiz reviewing material from the previous class; in an advanced class, this quiz may be replaced with an out-of-class assignment on material from the previous class or on prerequisite material from previous courses. Student groups then progress through the activity and write down a common set of answers to a series of questions crafted to elicit inferences and conclusions. Application questions may be of the end-of-chapter type, or they may be more conceptual than computational. The recorder may turn in a copy of the activity with the group's answers at the end of the period, or may record the important concepts that have been developed that day. A general chemistry text for POGIL has been published [53], as have many activity books geared toward various branches of chemistry [54–60]. Sample activities on a wide variety of topics may be downloaded free of charge from the POGIL website [13].

POGIL materials for some advanced courses may be different. Many of the concepts for an advanced course (such as biochemistry) have been introduced in prior courses and concept invention is not needed. Still, students must make connections that include the prior knowledge and extend that knowledge by examining new relationships involving these previously encountered concepts. Thus, the questions that comprise the exploration phase and concept invention phase of the learning cycle may be somewhat different in advanced courses compared with those in an introductory course, even though their purposes remain consistent with the learning cycle approach.

All of the PX_n_L pedagogies confront the learning process in which students periodically reflect on what they learned, how they learned it, and what works best. This metacognitive approach was prominent in the original medical school PBL model and is now especially emphasized in POGIL.

## TEXTBOOKS AND OTHER RESOURCES

Because PLTL and POGIL have a distinctly disciplinary focus, they use a textbook. However, the text is used differently. With PLTL, the course includes lectures and a structure that often follows the sequence of chapters in a textbook. Students are expected to read the textbook before coming to lecture and workshop, complete assigned problems from the text, and use the textbook during the PLTL workshop. For many introductory POGIL courses the textbook is not used during the class; it serves as a source of problems and a reference to consult on an as-needed basis after the concepts have been developed in class [16,61], whereas for advanced courses the textbook may serve as a source of models, graphs, and text used during the exploration and concept formation phase.

By contrast, the interdisciplinary and integrated nature of PBL means that topics are not limited to a single text or to a particular order of chapters. Students need to identify what they need to learn, look it up wherever they can, and be able to judge reliable sources. Textbooks, including those from previous courses, serve as resources along with the Internet and library resources. Often a collection of faculty desk copies serve as a classroom resource.

## ASSESSMENT AND GRADING

If the learning goals and the teaching and learning activities of a course change, but the assessment of students' knowledge does not change accordingly, a significant problem exists in course integration [62]. All of the PX_n_L pedagogies emphasize communication of conceptual understanding of course content. Consequently, student assessment must evaluate conceptual understanding over rote memorization. Students need to demonstrate their ability to use what they know by processing and evaluating information. They need to be able to explain what they know clearly and in complete sentences. While most instructors limit their use of multiple-choice tests for PBL [63], PLTL, and POGIL, there are ways to incorporate appropriately designed multiple-choice tests effectively and remain consistent to the philosophy [64–66].

In addition, all of the PX_n_L pedagogies involve group work in which individuals have responsibility to group practice and function. This can be done with peer and self evaluations, quizzes and examinations, and by facilitator observations. While these assessments of attendance, participation, preparation, and attitude may only be a small part of the student's grade (≤10%), ignoring these issues implies they are not valued [19]. For example, some PLTL courses assign modest credit for participation in the workshop.

Although not commonly used, some intensive PBL courses employ group parts for 25% of midterm and final examinations [67]. For POGIL in particular, the use of personal response systems (“clickers”) is also showing great promise for assessing student comprehension, for pacing classes of any size, and as a means for easily assigning participation credit [68].

## SCALABILITY

Because many science courses involve large lecture classes, the additional logistics of creating, scheduling, supervising, monitoring, and training associated with cooperative learning groups in PX_n_L courses make scalability a significant issue. Of the three pedagogies, PLTL emerged in a large class setting. The lecture is retained and the workshops meet at separate times in smaller settings. The size of the class is only limited by support for workshop leaders and the availability of meeting rooms. PBL, as it developed in medical schools, was intended to replace lectures and have each tutorial group of eight to ten students meet with one facilitator from a cadre of clinical or basic science faculty facilitators. Because most colleges and universities lack such extensive instructional resources, multiple smaller PBL groups often meet together with the instructor as a floating facilitator during the scheduled “lecture” time. The groups may also include peer facilitators. In the absence of dedicated group facilitators, students will often have assigned roles in PBL groups much as in POGIL. Larger classes with more than 35 students or more than five to seven PBL groups, and non-ideal teaching spaces can be accommodated, but these require more structure and planning, sometimes tending toward the POGIL model [69].

Similarly to PBL, POGIL groups meet together in the same room with a single instructor serving all groups. Because POGIL has a rather structured format with multiple groups working simultaneously on the same tasks, some scale-up is possible with a corresponding sacrifice in the group contributions to whole class reporting and extra demands on monitoring and attending to the needs of individual groups. Still, there are a number of strategies that have been successfully used for implementing POGIL in large classrooms, including the use of clickers, as mentioned in the previous section [70].

## OUTCOMES ASSESSMENT

The PX_n_L pedagogies fall in the categories of active and cooperative learning. Of the assessment of the educational effectiveness of these approaches, Richard Felder states:

Such teacher-centered instructional methods [traditional lectures] have repeatedly been found inferior to instruction that involves active learning, in which students solve problems, answer questions, formulate questions of their own, discuss, explain, debate, or brainstorm during class, and cooperative learning, in which students work in teams on problems and projects under conditions that assure both positive interdependence and individual accountability. This conclusion applies whether the assessment measure is short-term mastery, long-term retention, or depth of understanding of course material, acquisition of critical thinking or creative problem-solving skills, formation of positive attitudes toward the subject being taught, or level of confidence in knowledge or skills [71].

Specific assessments of PBL, PLTL, and POGIL support such claims, although the individual studies vary as conducted by different instructors in different disciplines. There is clear evidence that POGIL improves performance in both organic and introductory chemistry in terms of increased attendance, better grades, decreased dropouts, and increased enrollment in subsequent chemistry courses [72–74]. Aside from issues of performance, students and instructors find the POGIL classroom environment enjoyable [13,75] and conducive to the development of important learning skills.

Like POGIL, data from numerous studies of PLTL instruction also show significant gains in performance, retention, perseverance, and student attitudes and opinions [74,76–82]. Concerns that group work comes at the expense of content have been dispelled by the performance of workshop students on standardized American Chemical Society examinations. Other reports anticipate not only improvements in grades for students in PLTL classes, but also gains in general intellectual process skills, higher-order thinking skills, and improved ability to talk about scientific concepts [83,84].

PBL as practiced in medical schools has been evaluated extensively [85], and the results are mixed. Although PBL students performed as well or better clinically and were more likely to enter family medicine, their performance on basic science examinations were sometimes lower. As with other forms of active learning, the students enjoyed the process more than traditional pedagogy. Among the perceived challenges for assessing PBL are its inherently interdisciplinary nature and its heavy emphasis on self-directed learning, which require different assessment instruments [86,87]. A standardized test on factual knowledge in a specific discipline might well yield poorer performance for PBL students when compared with students taking traditional courses whose major focus is on content. Consequently, most PBL instructors are satisfied when assessments show little or no difference in content knowledge. What they would really like are assessment instruments that could document the improvement in students' ability to learn on their own—their growth in intellectual maturity that translates into taking personal responsibility for learning throughout their lives. This is a major emphasis for PBL and a reason many instructors are attracted to it. Generally, PBL requires a greater level of intellectual maturity on the part of the students than either POGIL or PLTL because PBL students are expected to generate their own questions to drive their learning, while the instructor's questions drive inquiry in both POGIL and PLTL.

## FACULTY AND STUDENT ACCEPTANCE

Rare is the teacher in higher education who has experienced any of the PX_n_L pedagogies as a student. Consequently, there is a significant barrier to acceptance and adoption of these unfamiliar methodologies that challenge personal beliefs. As highlighted recently [88], this results in a gap between what is now known about learning and the way science is often taught. This is a major concern of those interested in reforming science education with the adoption of active learning strategies, as the following question indicates:

So why do outstanding scientists, who demand rigorous proof for scientific assertions in their research, continue to use and, indeed, defend on the basis of their intuition alone, teaching methods that are not the most effective? [89]

Faculty development workshops address issues of resistance and put participants in the role of learners. Nevertheless, such experiential approaches, anecdotal testimonies and even data about efficacy encounter skepticism and resistance. Even when faculty accepts that active-learning methods have merit, there is an activation energy to try something new and a fear of the consequences of failure [90]. Adoption of a different pedagogy also carries with it a many unexpected new practical and organizational issues [91]. Of the three PX_n_L pedagogies, PLTL generally involves the least and PBL the greatest departure from traditional instruction [92]. However, in schools without recitations, adding a 2-hour “workshop” to a course can be challenging. Likewise, to the extent that serious leader-training is undertaken, an additional set of resources employing learning specialists may be daunting. Nonetheless, faculty who embrace the need for instructional change and conquer the initial steep learning curve find the experience transformative.

There is work in adopting these new pedagogies. Fortunately, much of the effort is startup; in the steady state, the faculty work load is similar to that in traditional courses. There are also some decreases in work load. Since all three methods emphasize the responsibility of the student for learning, office hour traffic from dependent students usually decreases. The developers of the methods have worked diligently to reduce the activation barriers for new adopters by documenting their methods and appropriate problems. The PLTL project has published a *Handbook for Team Leaders* [93] for use in leader training courses. A recent research paper describes new dimensions in leader training for PLTL [94]. The POGIL website provides information concerning implementation in the classroom, including the *Instructor's Guide to Process-Oriented Guided Inquiry Learning*, which can be downloaded at no cost [19]. In addition, an ACS symposium series devoted to POGIL is available and includes the topics of implementation strategies in a variety of contexts and effectiveness [95] and several sources for general information about POGIL [16,96].

As a general rule, students prefer the interactive format of active-learning pedagogies and they appreciate being challenged [97,98]. Key elements in assuring acceptance from students include clear explanation of the classroom format and expectations, an understanding of how the format is connected to research on learning, and frequent reinforcement of how the classroom activities will benefit them.

Much of the early research and development work on PLTL was driven forward by the overwhelming enthusiasm of students and peer-leaders for the new format [28]. Student testimony is powerful and often converts skeptics who are not convinced by faculty and staff innovators. Peer leader opinion is even more forceful because the peer leaders have a unique perspective on the course and are among the best students in the institution.

A universally recognized benefit of active-learning pedagogies that use peer facilitators is the effect of the experience on the facilitators [38,99–101]. They, perhaps more than the students they serve, benefit from the experience. Not only do they consolidate their disciplinary understanding, they often develop a mentoring relationship with faculty instructors and thereby gain considerable insight into the teaching-learning process and enterprise. They often report that they have a new perspective on themselves and how they approach learning in all of their courses. Some decide to become teachers. All leaders report significant gains in their understanding of others and in a supporting portfolio of marketable skills such as leadership, communication, and team-building. These benefits are most apparent in the PLTL pedagogy because it is built around the power of the peer leader and emphasizes structured peer leader training.

A recent multi-institutional study of student perspectives on the use of POGIL in organic chemistry showed overwhelmingly positive attitudes, with fewer than 8% of more than 1,000 students being negative about the method [102], when compared with 30% who expressed negative attitudes toward the traditional lecture approach [13]. Students' assessment of their own growth in process skills in organic chemistry were measured using the Student Assessment of Learning Gains survey [103]. The students experiencing a POGIL approach in class reported significantly higher gains in their own process skills compared with those students whose classes were taught in a lecture format.

## HYBRIDIZATION AMONG PEDAGOGIES

Much of the confusion associated with labels for different pedagogies is that individual instructors adopt and adapt instructional ideas that suit them and their situation [104]. As a consequence, the archetypical models of PBL, POGIL, or PLTL often become blended with each other and with other pedagogies to the point that one short acronym is insufficient to capture what goes on in the classroom. For example, PBL as originally conceived expected students to define what they needed to learn. The students then spent considerable time outside of class locating and studying what they found in order to share it in class with others in their group. However, much of what instructors now call PBL has a significant guided-inquiry component and the problems, though retaining their real-world relevancy, can be completed in class with textbooks and other resources. Such modifications may be quite appropriate for the situation though they are probably indistinguishable from pedagogies associated with case studies [105]. Similarly, those POGIL courses that devote more class time to application acquire some of the characteristics of PBL courses.

Some instructors deliberately hybridize different pedagogies with the intent of optimizing the beneficial elements of each. For example, peer-led guided inquiry (PLGI) combines elements of POGIL and PLTL [74]. The approach is based on POGIL materials, but employs peers as facilitators, as in PLTL. In contrast to POGIL, which dispenses with regular lectures entirely, the PLGI workshop sessions replace only one of three weekly lecture sessions, and thus resemble a common variant of PLTL in which workshops are conducted once per week in place of the lecture.

## PEDAGOGIES OF ENGAGEMENT

Studies of how people learn show that active-learning environments involving problem-solving discussions with peers are more effective than traditional lectures. There is no doubt that good lectures are efficient ways to illuminate course content and that they work quite well for some students, but even the best lectures remain generally in the realm of “passive learning,” and it is arguable that some students survive despite this approach, rather than because of it. Moreover, the contention that “if it ain't broke, don't fix it,” flies in the face of the many studies (NSF and otherwise), indicating that a large fraction of students are not served well by this traditional model, and may be hindered by the “one size fits all” model of higher education, contributing to the demonstrated attrition of underrepresented groups in the STEM disciplines. In contrast, the evidence is that all students thrive when their education is supplemented with the structured interdependent settings created by various PX_n_L pedagogies. Our goal here is to outline the resource- and implementation-dependent nuances and variation available within this subset of pedagogies. We hope that our comparisons will provide the impetus for the adoption of significant improvements to our current educational system, which Ibarra (2001) argues,

… is literally teaching only half the knowledge base—the information that tends to be readily absorbed by roughly half of the population—and it continues to do so with only half the information about learning methodology and pedagogy currently available to it [106].

The dilemma of serving the wide variation in today's constituency of learners has been examined in the context of a PBL approach, using peer leaders and applying Ibarra's ideas of a “multicontextual” learning environment [107]. This means deliberately maximizing the accessibility to learning by all kinds of students, using a variety of styles and approaches such that everyone can benefit optimally.

All of the authors of this paper have years of experience teaching in a lecture format and most still enjoy lecturing. However, we have discovered that there is a difference when one teaches beyond a content-driven curriculum towards the goals of student learning and understanding. For that reason, we teach differently than we were taught and encourage readers, who wish to do the same, to attend to the literature and any of the several dissemination workshops for PBL [108], PLTL [109], or POGIL [110].

## The abbreviations used are:

PBL: problem-based learning
POGIL: process-oriented guided inquiry learning
PLTL: peer-led team learning
PLGI: peer-led guided inquiry
